# A Perspective to Control Laser-Induced Periodic Surface Structure Formation at Glancing-Incident Femtosecond Laser-Processed Surfaces

**DOI:** 10.1007/s11837-021-04963-w

**Published:** 2021-11-01

**Authors:** Alexander Jelinek, Manuel J. Pfeifenberger, Reinhard Pippan, Daniel Kiener

**Affiliations:** 1 Department of Materials Science, Chair of Materials Physics, Montanuniversität Leoben, Franz-Josef-Straße 18, 8700 Leoben, Austria; 2 Erich Schmid Institute for Materials Science, Austrian Academy of Sciences, Jahnstraße 12, 8700 Leoben, Austria

## Abstract

The favorable combination of high material removal rate and low influence on the material beneath the ultra-short pulsed laser-processed surface are of particular advantage for sample preparation. This is especially true at the micrometer scale or for the pre-preparation for a subsequent focused ion beam milling process. Specific surface features, the laser-induced periodic surface structures, are generated on femtosecond laser-irradiated surfaces in most cases, which pose an issue for surface-sensitive mechanical testing or microstructural investigations. This work strives for an approach to enhance the surface quality of glancing-incident laser-processed surfaces on the model material copper with two distinctly different grain sizes. A new generalized perspective is presented, in which optimized parameter selection serves to counteract the formation of the laser-induced periodic surface structures, enabling, for example, grain orientation mapping directly on femtosecond laser processed surfaces.

**Supplementary Information:**

The online version contains supplementary material available at 10.1007/s11837-021-04963-w.

## Introduction

Ultra-short pulsed (USP) lasers have become a favorable tool in material science in recent years, since technological advances lowered hardware costs, such that these lasers found widespread application for surface modification and advanced laser cutting of various materials.^1^ Systems for material processing, regarding precise ablation, in combination with analytical techniques, enable a localized analysis of materials with high resolution and accuracy.^2^ Notably, even systems without built-in analytical devices,^3^ find application due to the high material removal rates in comparison to alternative techniques, e.g., focused ion beam (FIB) milling,^4,5^ making them beneficial for small-scale preparation tasks.

The preparation of micro-mechanical samples commonly involves laborious work steps and may consume noticeable amounts of working hours for a limited number of specimens. With the combination of well-established mechanical pre-preparation and other high ablation methods, such as electrode discharge milling or ion slicing,^6^ prior to high precision methods, commonly FIB milling, the preparation time is reduced considerably. This is especially true for specimens with dimensions in the range of tens to hundreds of micrometers. USP lasers, therefore, open completely new possibilities for the field of micro-fabrication,^2,4,7^ where a high ablation rate,^8–11^ in combination with form-free milling and absolute precision in the ten-micrometer range, allows closing the gap between the pre-preparation and the final manufacturing of a testing geometry. Precise and fast specimen preparation even allows for a statistically-based material assessment on the micro-scale.^12^

A finishing step is generally required, as the surface quality resulting from USP laser processing, as well as near-surface material modification, impedes surface sensitive analysis such as electron backscatter diffraction (EBSD) and might influence the results of mechanical experiments, especially regarding miniaturized samples. Although special cases might not pose such strict surface quality requirements, e.g., miniaturized fracture mechanical experiments,^13,14^ where a FIB notch acts as a defined defect, in general, FIB polishing is required to produce a diminishingly influenced and smooth surface before mechanical testing.

For USP laser-ablated surfaces, various side effects might occur. Due to the unique material ablation processes,^15^ a thin top layer of amorphized material with a thickness of tens of nm is formed. Beneath this, a less heavily affected zone of a few nm up to µm remains, strongly dependent on the material being investigated.^16^ The ablation process itself is hardly sensitive to the chemical character within the bulk,^17^ but topological effects can lead to the formation of cone structures, consisting of debris and unablated material underneath,^18,19^ which develop randomly distributed across the processing site. Further, unique surface features formed upon USP laser irradiation are the so-called laser-induced periodic surface structures (LIPSS).^20^ Their formation process involves an interaction of the electron plasma with the electromagnetic field near the surface during the USP laser irradiation, leading to interference between surface plasmons.^21^ LIPSS occur in two distinct shapes, being the low- and the high spatial frequency LIPSS, which appear perpendicular and parallel to the polarization direction of the laser, respectively.^22^ Here, the low spatial frequency shape is of special interest for sample preparation due to their continuous ripple-like appearance with height and periodicity in the range of a few hundred nm.

Regarding the LIPSS formation, many publications have dealt with processing parameters aiming to optimize their shape to utilize these features on various materials for surface patterning applications,^23–31^ even including polarization-dependent appearance.^32–35^ For this purpose, incident laser pulses perpendicular to the sample surface are commonly used. Notably, only a few references are available for the case of glancing incident, as commonly utilized for micro-fabrication.

Sikora et al.^7^ investigated the optimization of picosecond pulsed laser processing including FIB polishing for the sample preparation of electronic components. The quality of trenches, cut in silicon and full integrated circuits, were evaluated regarding the effect of laser processing parameters on the evolution of the sidewall inclination and the surface roughness. A higher fluence tends to increase the sidewall taper at an equal scan number, but a similar saturation taper is present for all fluences. Echlin et al.^16^ performed a detailed in-depth investigation of the femtosecond laser-processed surface on various materials to evaluate the influence of processing on the respective material surface, including EBSD techniques and transmission electron microscopy (TEM). In this study, the development of LIPSS, as well as material damage, has been observed for copper, strontium titanate, a nickel alloy René 88DT, {111}-oriented single crystal silicon, and gallium nitride. Precise FIB milling was applied for polishing and the preparation of cross-sectional TEM lift-out samples. For the case of coarse-grained (CG) copper, dislocation damage was found up to 5 µm in depth, along with crystallographic rotation to depths of 1–4 µm, and local regions of up to 2 µm with misorientations of 4°–7° compared to surrounding grains on the surface.

Nevertheless, if the formation of LIPSS could be avoided for microscopic investigations, and in general for miniaturized sample preparation, major FIB milling efforts could be reduced or even completely avoided. Therefore, the aim of this study is to compare processing conditions to obtain optimized residual surface quality. A broad processing parameter variation of a reproducible trench geometry was conducted on pure copper. Subsequent, surface investigations were performed to illuminate the individual effects. Based on this experimental input, a model covering the fundamental parameters of the ablation process^23,36^ and surface structuring,^23,24,37,38^ being fluence, scanning speed, pulse repetition rate, and repetitions of the scanning pattern, is presented. Furthermore, the formation of LIPSS on glancing-incident-processed surfaces is discussed in terms of surface roughness. Here, an EBSD investigation is performed as an indicator for the surface quality and to demonstrate the possibilities for optimized laser processing parameters for micro-mechanical preparation applications.

## Experimental

### Material

Technically pure copper (99.99%) with a grain size of ~ 50 µm was cut to dimensions of about 2.0 × 4.5 × 20.0 mm using a Secotom precision cutting machine. The resulting samples were then electrolytically polished on two perpendicular faces to achieve a defined edge for laser processing experiments. Furthermore, ultra-fine grained (UFG) copper obtained by high pressure torsion^39^ with 10 rotations at a nominal pressure of 2.8 GPa was prepared equally from disks, with a diameter of 30 mm and thickness of 6 mm. Owing to the resulting grain size of ~ 500 nm, this sample allowed to study the influence of grain size with respect to the laser-ablated surface roughness regarding EBSD image quality.

### Material Processing Pattern and Parameters

The laser processing was conducted with a scanning electron microscope (SEM)/FIB platform (Auriga, Carl Zeiss, Oberkochen, Germany) equipped with a femtosecond laser (Origami 10 XP; Onefive, Regensdorf, Switzerland). The wavelength was set to 515 nm and pulses with a duration of 318 fs were applied at a base frequency of 50 kHz. All the processing steps were conducted under vacuum conditions. The focal point was found manually by varying the distance between the dispersal and collecting lenses along the optical path of the beam expander of the system and was kept constant during each processing experiment. The diameter *D* of the focused laser spot was previously determined to be about 24.5 µm. For further details on the measurement set-up, the reader is referred to Ref. 4.

**Fig. 1:**
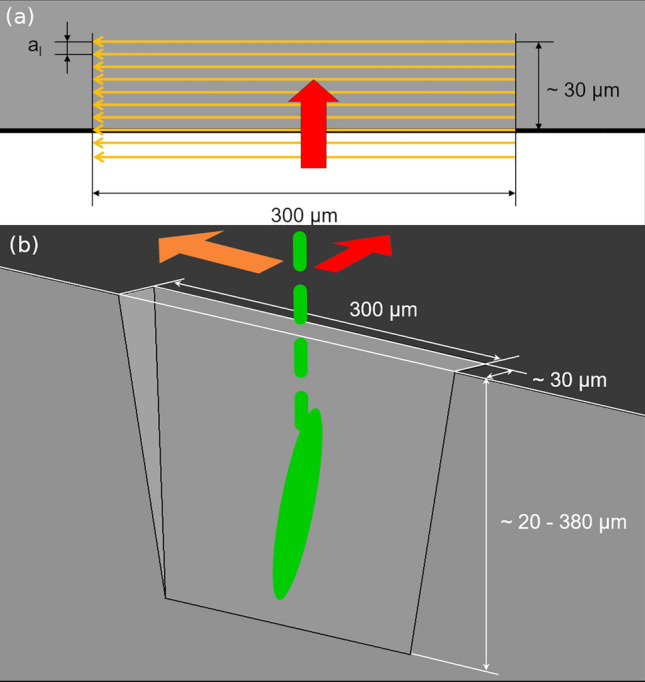
(a) Top view, in the laser beam propagation direction, of the processing pattern to obtain trenches for subsequent surface quality analysis. The field in *gray* represents the bulk material, where the *thick black line* depicts the sample edge. Unidirectional laser spot translation is indicated (*thin orange arrows*), which are stepwise parallel-shifted towards the sample center (*thick* red arrow) by *a*_l_. (b) Idealized trench obtained by the processing pattern indicated in (a). Laser pulses (*dashed green line*) reaching the material surface causing stepwise ablation of the material, leaving a strongly inclined surface, where the projected pulse area (*green oval*) increases (Color figure online).

The processing parameters, such as continuous output power *P* and divisor *div,* were varied throughout the experiments. The divisor, a laser device-specific feature, enables the blocking of a certain fraction of pulses and thereby adjusts the pulse frequency. The base frequency *f*, which defines the pulse energy, is not influenced by the divisor. Since the focus of this work lies on glancing-incident laser processing on bulk materials, a simple pattern was chosen to mimic conventional cutting processes, which furthermore allows good comparability with other systems. The processing pattern is illustrated in Fig. 1a and consists of parallel, unidirectional lines with equal length and a certain intermediate spacing *a*_l_. Further processing parameters of the pattern are the number of individual line repetitions *l*, the repetitions of the whole pattern *scans*, and the scanning speed *s,* being the translation speed of the laser focal spot. All relevant parameters are listed in Table I.

**Table I:** Relevant fixed and varied laser processing parameters used in this study

Fixed parameters	Abbreviation	Value/Common unit	Description
Base Frequency	*f*	50 kHz	Base pulse repetition rate/frequency
Wavelength	*λ*	515 nm	Wavelength of laser
Spot diameter	*D*	24.5 µm	Laser spot diameter on the sample surface

Varied parameters		Common unit	
Divisor	*div*	–	Defines effective pulse rate
Power	*P*	mW	Continuous output power
Lines	*l*	–	Repetition of single scan lines
Scans	*scans*	–	Repetition of the processing pattern
Line distance	*a*_l_	µm	Distance between parallel lines
Scanning speed	*s*	mm/s	Speed of the laser spot

The pattern was placed near the polished edge of the bulk material, resulting in an idealized trench of about 300 µm length and about 30 µm depth. For the case of actual trenches, the Gaussian intensity distribution along the pulse diameter leads to low ablation in the outermost regions of the processing site, whereby the dimensions appear to be enlarged by the spot diameter *D* and the formation of a sharp edge is inhibited. Furthermore, the trench depth underlies the absolute positioning precision of about 10 µm, although the relative precision is below 1 µm.^4^ The actual trench height also varies according to the applied parameter combination between 20 µm and 380 µm.

The laser processing was performed on the edge of a sample to minimize the redeposition of debris and to facilitate subsequent surface analysis, as illustrated in Fig. 1b. During removal of the material, the laser spot becomes increasingly elongated (as depicted in Fig. 1b), due to the projection on the inclined trench surface, which dominates the defocus error. Due to this geometrical effect, the laser spot area increases while decreasing the local energy density of the individual pulse. The consequences of this will be discussed later.

For the description and understanding of the ablation process, the interplay of geometrical parameters and pulse properties are taken into consideration. The laser spot area *A*_*D*_ was approximated as a perfect circle, and, together with the base frequency *f,* the fluence $\Phi$ is obtained by applying Eq. 1. Based on the applied individual continuous power values *P*, the fluence represents the peak areal energy density of a pulse at the focal spot. The factor of two originates from the gaussian intensity distribution^40^ along the spot diameter, where a flat-top approach would omit this factor.

1 $$\Phi =\frac{P*2}{f*{A}_{D}}$$

For sake of completeness and further considerations, the distance between two subsequent pulses *a*_*P*_ is introduced by Eq. 2, where the scanning speed *s*, the devisor *div*, and the base frequency *f* are included.

2 $${a}_{P}= \frac{s*\mathrm{div}}{f}$$

A detailed list of all the parameter combinations applied along all the processed trenches can be found in the supplementary material, Table S1.

### Surface Investigation

A field-emission SEM (Auriga; Carl Zeiss) was utilized to acquire images (acceleration voltage: 5 kV, secondary electron detector, working distance: ~ 20 mm) of each trench with different magnifications for a visual qualification regarding the development of LIPSS. In addition, quantitative surface roughness measurements were conducted on selected trenches with a confocal laser scanning microscope (CLSM; LEXT OLS 4100; Olympus Europe, Hamburg, Germany) and corresponding evaluation software (v.3.1.9.2). The CLSM scans were performed with the trench surface facing up and a ×100 objective. A three-spot compensation was applied to achieve a horizontal height profile and eliminate possible variations in trench taper. Generally, a measurement window of 1024 × 1024 pixel (129 × 129 µm) was used. At certain trenches, only a smaller area was available, and the window was thus reduced to 512 × 512 pixel (65 × 65 µm). Via visual cross-validation, the arithmetic mean height *S*_a_ of the surface, according to ISO 25178, was determined to give the most representative value for the remaining wavy surface, and therefore the amount of generated LIPSS was quantified on the base of roughness. Representative CLSM images regarding the surface roughness measurements for trenches processed at different fluence levels and with different geometrical processing conditions, can be found in the supplementary material Figs. S1–S5.

Finally, EBSD scans were conducted to demonstrate the influence on surfaces quality, obtained by different processing parameters, regarding the image quality of grain orientation maps. For this a field-emission SEM (LEO 1525; Carl Zeiss) operated at an acceleration voltage of 20 kV and an EBSD detector (Ametek; EDAX, Mahwah, NJ, USA) were used, whereby the data were analyzed with the software TSL OIM Data Collection v.5 and for post-analysis TSL OIM Analysis 5.31. For the mapping on CG copper, an imaging step size of 200 nm was utilized. To further outline possibilities and limitations of surface-sensitive EBSD analysis directly on laser-processed surfaces, also UFG copper was prepared with two selected sets of processing parameters. For this material, an imaging step size of 20 nm was chosen to account for the smaller grain size of ~ 500 nm.

## Results

### SEM Characterization

**Fig. 2:**
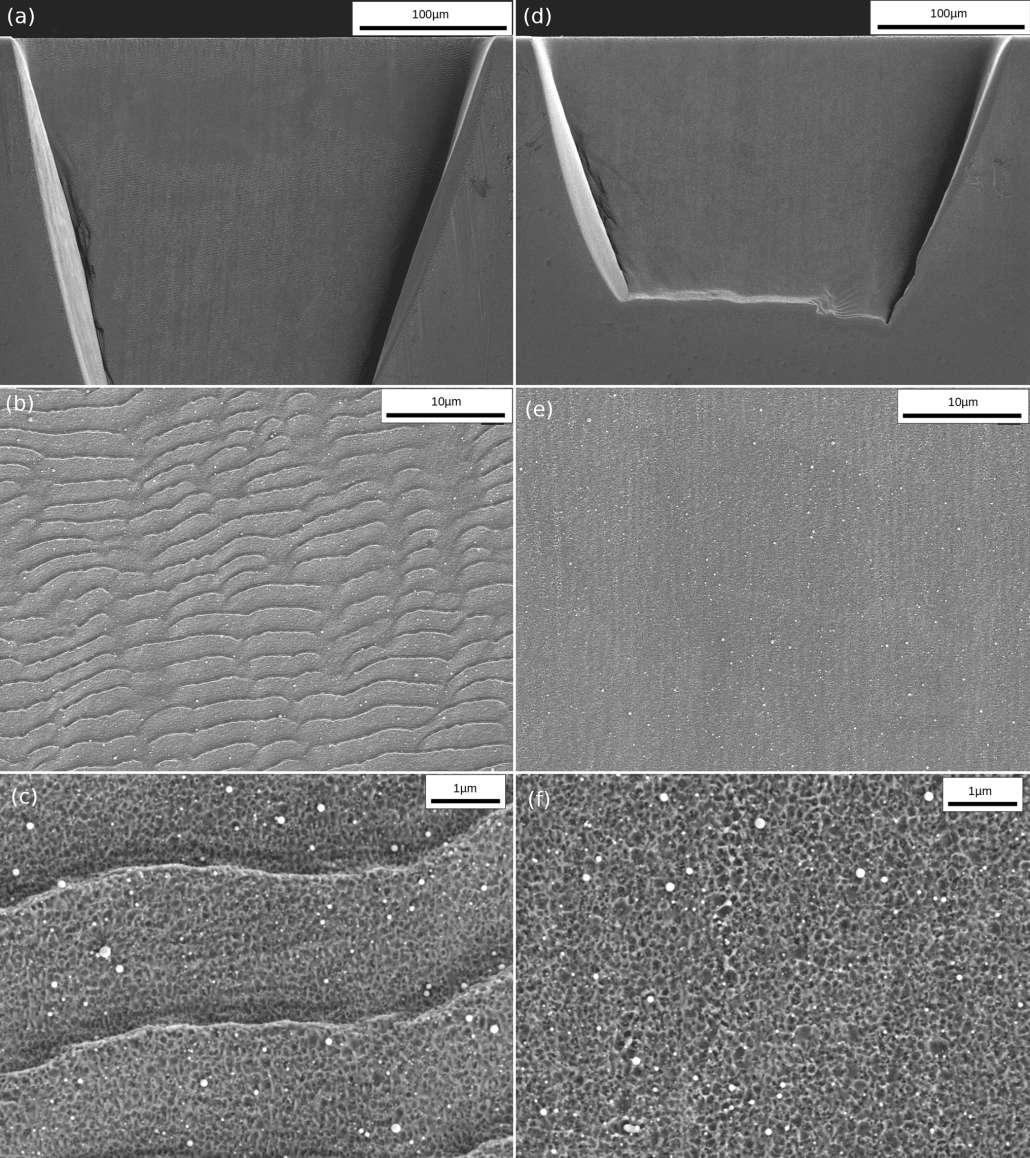
(a–c) Laser-processed trench in CG copper with increasing SEM magnification (*Φ* = 1.96 J/cm^2^, *div* = 50, *s* = 1 mm/s, *l* = 3, *scans* = 100 and *a*_l_ = 5 µm). LIPSS, well distinguished in (b) and detailed in (c), fully cover the trench indicated in (a). (d–f) Trench without LIPSS at identical SEM magnification steps as in (a–c) (*Φ* = 1.96 J/cm^2^, *div* = 50, *s* = 1 mm/s, *l* = 1, *scans* = 50 and *a*_l_ = 5 µm). In (c) a highly magnified image of the LIPSS is shown, which are covered with ablated surface, while in (f) only this last type of micro-roughness in the 10-nm regime is present.

A classification of the surface regarding LIPSS formation was applied on every trench via SEM images. In Fig. 2a–c, a representative trench, covered with LIPSS over a large fraction of the surface area is shown with different magnifications to visualize the surface topography at different length scales. The processing parameters can be found in the image caption. In Fig. 2d–f, a trench with different parameters is illustrated at equal magnification steps, where no LIPSS are present. Figure 2b shows LIPSS in detail, where the strongly inclined surface concerning the laser irradiation direction leads to a relative increase in their periodicity compared to a flat formation, where the periodicity should be about 420 nm at the present wavelength.^37^ The appearance itself is in strong contrast to Fig. 2e, where no LIPSS are visible at all. For the case of higher magnifications in Fig. 2c and f, the micro-roughness obtained by the ablation process is evidenced, along with redeposited material in the shape of small spheres.

### Pulses Per Spot PPS

Starting from a broad variation of parameters, with the intent to perform a screening for possible influences, a pulses per spot (PPS) related description of the processing pattern was derived, inspired by earlier works.^23,41^ For scanning a certain area with a laser spot, a parameter describing the number of laser pulses each point is exposed and can be defined by Eq. 3 and directly expressed in terms of processing parameters (see Table I) employing Eq. 2:

3 $$ {\text{PPS}} = \frac{D}{{a_{P} }} \times \frac{D}{{a_{l} }} \times l \times {\text{scans}} = \frac{D \times f}{{s \times {\text{div}}}} \times \frac{D}{{a_{l} }}  \times l \times {\text{scans}} $$

The first two terms in Eq. 3 represent the laser pulse overlap in line $\frac{D}{{a}_{P}}$ and between neighboring lines $\frac{D}{{a}_{l}}$, followed by the repetition number of lines *l* and pattern *scans*, respectively. Due to a comparatively large spot diameter *D* of 24.5 µm versus an *a*_*P*_ smaller than 7 µm and *a*_l_ smaller than 10 µm, the overlap coefficients were always larger than 2.5, indicating strong pulse overlap in and between lines. According to that, the actual Gaussian shape of the energy distribution of the laser spot is neglected. Thus, this description will be invalid at the border of the pattern, where edge effects due to the Gaussian tails of the laser spot dominate.

### LIPSS Versus Fluence and PPS

**Fig. 3:**
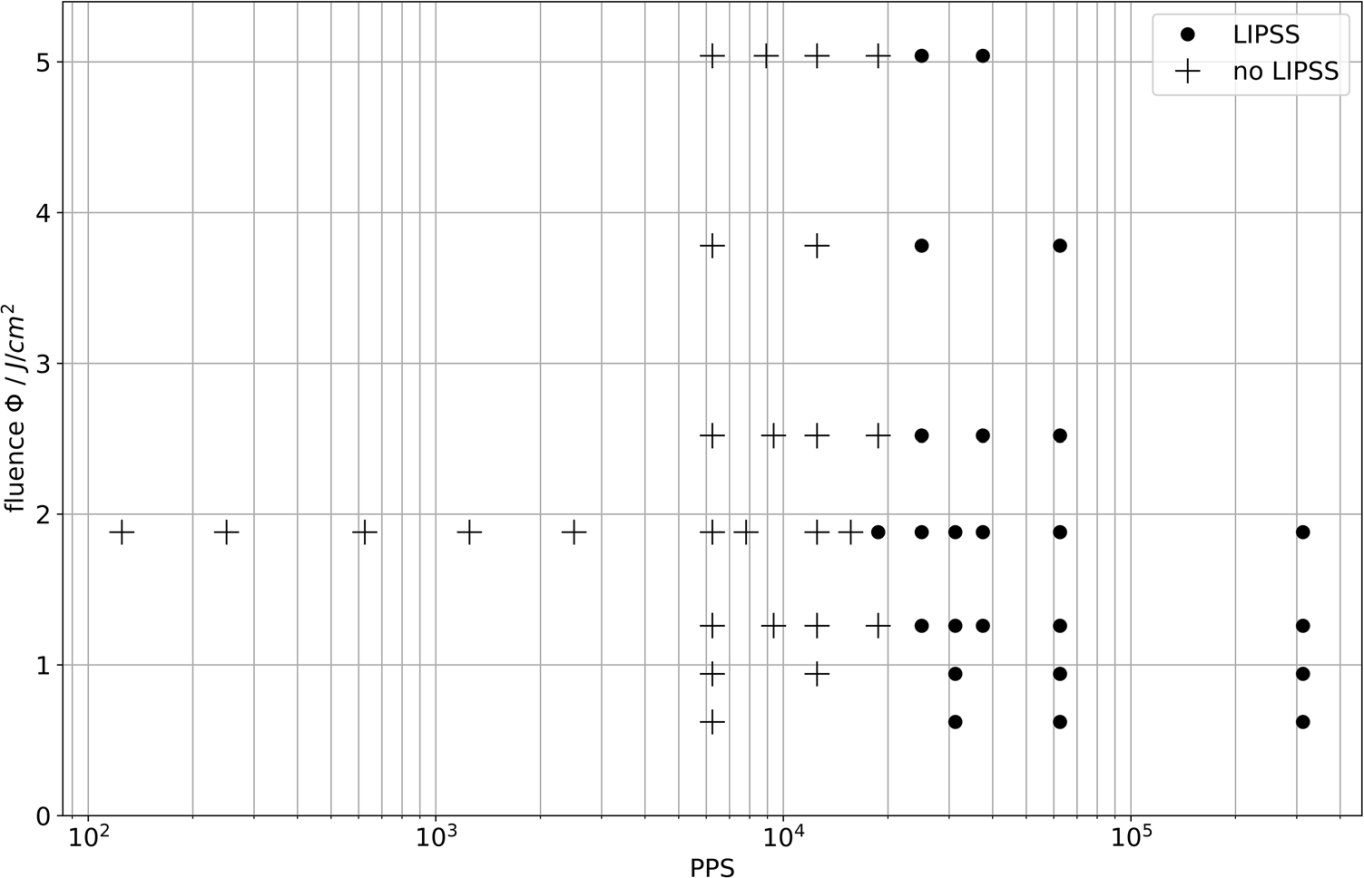
Pulses per spot (PPS) versus fluence of each trench investigated. The *points* are marked with respect to the qualitative appearance of LIPSS. If even a small area of LIPSS formed, the parameter combination is marked with a *black dot*, otherwise, the parameter combination is denoted by a *cross*.

The result of the first qualitative observation over a large parameter window was interpreted in terms of PPS and is illustrated in Fig. 3, where the fluence level is plotted over the semi-logarithmic PPS count. A combination was marked negative (black dot), if any LIPSS were observed on the surface, and positive (cross), if no LIPSS developed. The upper edge of the trenches was neglected, because the PPS model is invalid there in any case. A PPS number of below 18 k appeared to be the onset for the formation of LIPSS on glancing-incident-processed surfaces, where no explicit fluence dependency seems to be present regarding the qualitative observation and applied levels.

However, from the examination of SEM images, a trend regarding the evolution of LIPSS was identified. After spot-wise nucleation taking place in separated areas, these areas increase in size as the PPS increase until the whole surface is covered. This general development was observed for all fluence levels. In the supplementary material, Figs. S1–S5, the evolution of LIPSS-covered areas is shown by CLSM images of trenches obtained at different fluence levels with increasing PPS.

### Roughness Investigation

**Fig. 4:**
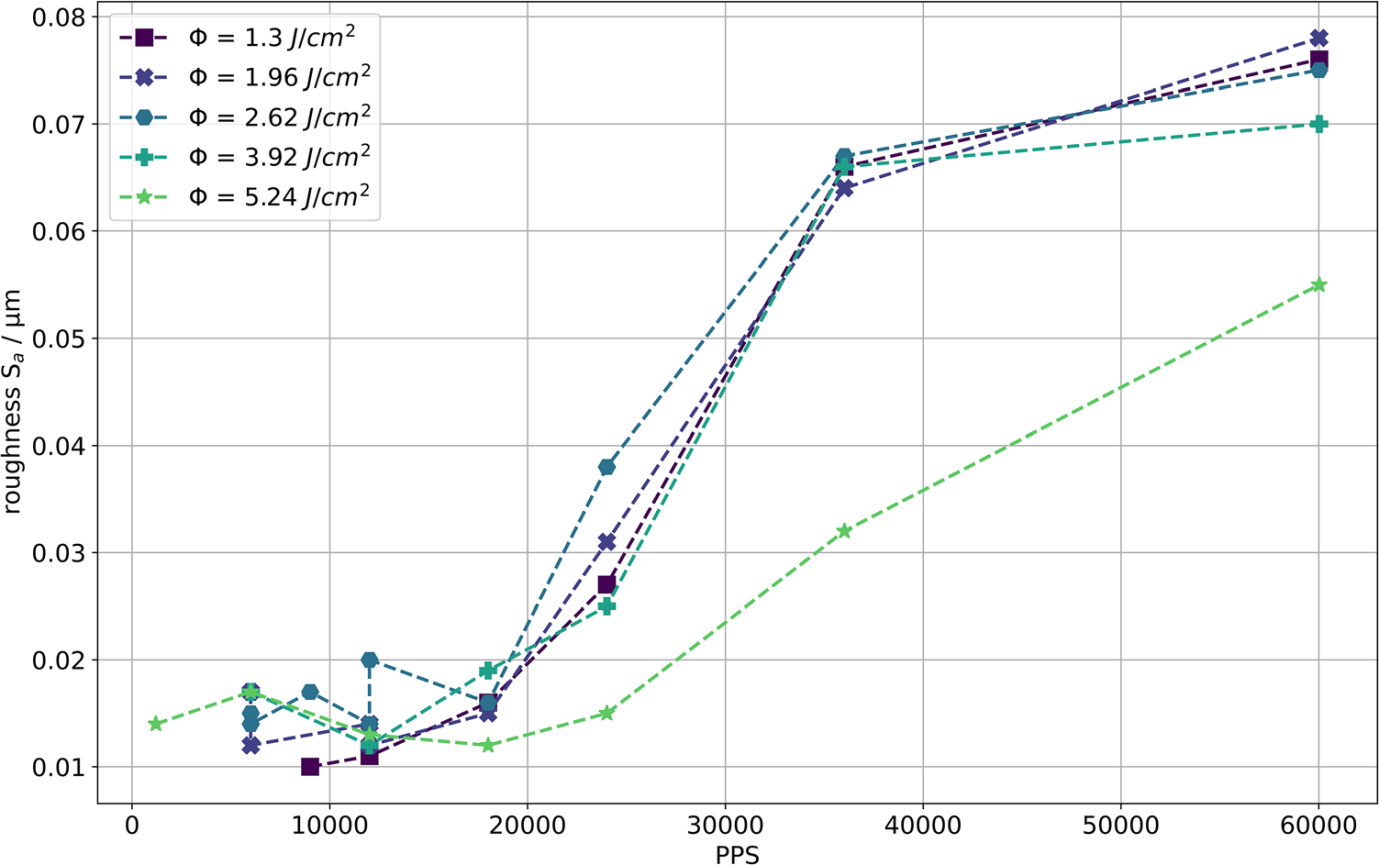
Evolution of average surface roughness with PPS for different fluence levels. The increase of roughness, with an onset at just below 18 k PPS, respectively 25 k, correlates with increasing areas covered with LIPSS. Once the whole region of interest is covered with LIPSS, the roughness saturates.

Via the height data of the CLSM image, further quantification of the LIPSS formation trend was performed. The roughness *S*_a_ was therefore plotted as a function of the PPS, as shown in Fig. 4. For all fluence levels, a nearly equivalent roughness trend is observed. A low roughness, associated with micro-roughness (see Fig. 2f) is present at a low PPS count, whereby the roughness starts to increase slightly below 18 k PPS, together with the appearance of LIPSS on the surface (see also supplementary Figs. S1–S5). The trends for all fluences saturate at about three to four times the base roughness value once the whole evaluated area is covered with LIPSS (see Fig. 2c). Only for the highest fluence level, the LIPSS-associated arithmetic mean height yields about half the values at equivalent PPS counts, whereby the imaged trend shows an equivalent but retarded evolution of LIPSS fields, with a nucleation threshold of slightly below 25 k.

Furthermore, the evolution of the trench taper was documented via vertical height profiles along with major parts of the processed surface. In the supplementary Fig. S6, the evolution of the taper profiles is illustrated for selected trenches processed with fluence levels of 1.96 J/cm^2^ and 5.24 J/cm^2^, respectively, and several different PPS values. In both cases, the trench taper increases with increasing PPS until a certain saturation angle of approximately 8°–10° is reached near 18 k PPS.

### EBSD Analysis

**Fig. 5:**
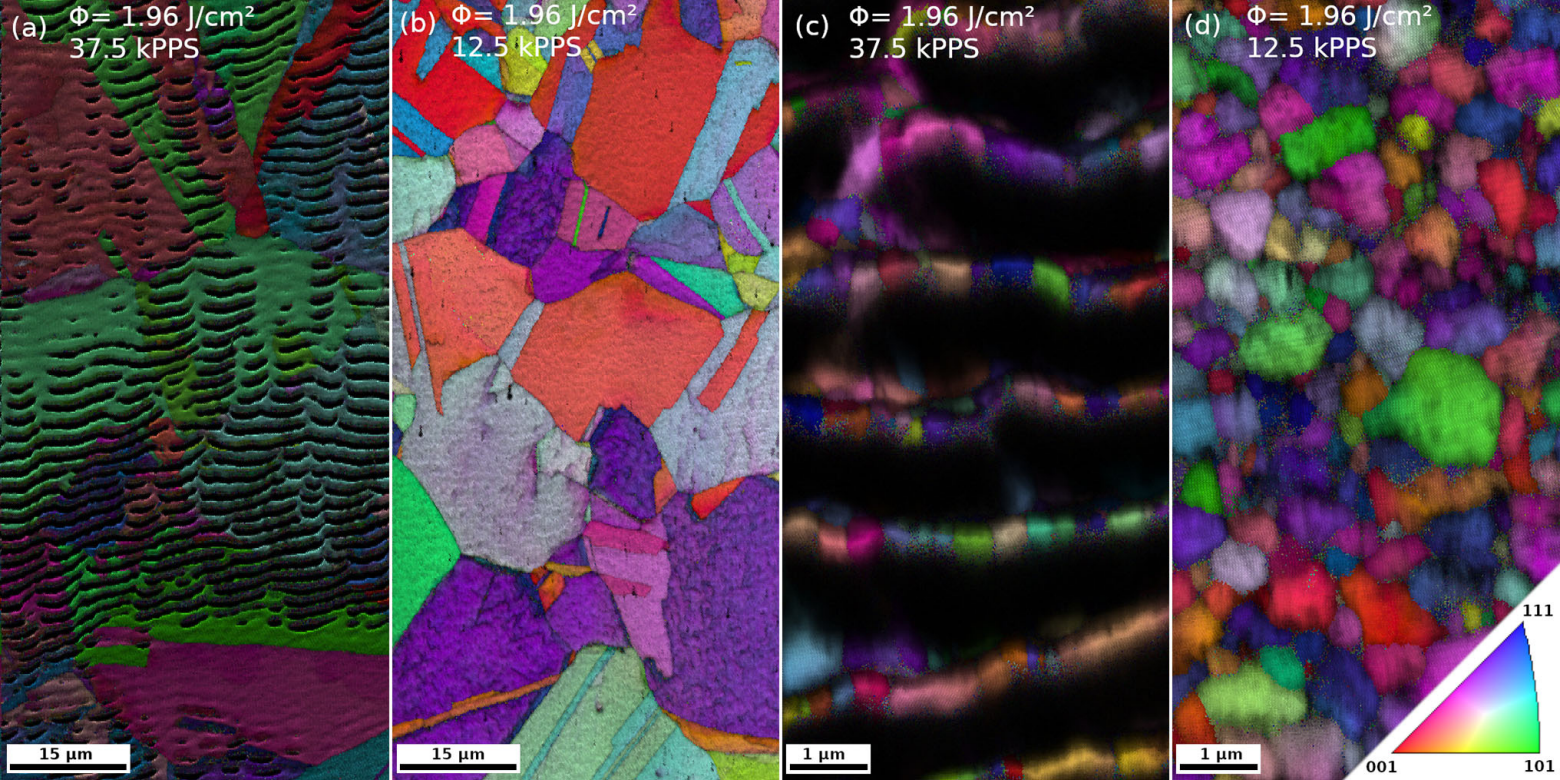
EBSD images (inverse pole figure with superimposed image quality) of CG copper (a, b) and UFG copper (c, d), whereby (a, c) are with and (b, d) without LIPSS according to the utilized parameter combination. On LIPSS-covered surfaces partly shadowing by the structures is evident, and an exact orientation mapping is hampered or even impossible. In the case of (a), single grains can be identified by crystallographic orientation, but a reliable mapping is not possible; even more so in (c), where the grain size is noticeably smaller than the LIPSS periodicity. On LIPSS-free surfaces in (b, d), EBSD scans can be reliably applied directly after laser processing. In the CG situation (b), a full scan is achieved, while in (d), even grains which are well below 500 nm in size can be identified.

For the final assessment, a damage and roughness sensitive analysis method, namely EBSD, was applied. Figure 5a–d illustrates EBSD-acquired inverse pole figure maps, which show the crystal orientation parallel to the viewing direction and the superimposed image quality of copper with two different grain sizes and two selected sets of processing parameters, below and above 18 k PPS, respectively, and at equal fluence levels. In Fig. 5a and c, the imaging quality is reduced due to shadowing by the LIPSS topography, so that no indexing can be performed within the valleys. In the case of Fig. 5a, the grain size is considerably larger than the LIPSS periodicity. Consequently, the hills of the LIPSS show equal orientation and, together with LIPSS-free areas, an overall impression of grain orientation remains possible. In the case of Fig. 5c, the grain size is smaller than the LIPSS periodicity, so that whole grains are shadowed within the valleys and a reliable orientation mapping cannot be performed anymore. In the optimized conditions of Fig. 5b the absence of LIPSS enables an orientation mapping with sharply defined grain boundaries, where the remaining low surface roughness enables to resolve even twins with thicknesses below 1 µm. Figure 5d shows the case of UFG copper of identical surface roughness but at a higher SEM magnification. The last figure shows a complete orientation map, and generally proves a clear qualification of small grains with diameters in the range of 500 nm. For grains smaller than that, the remaining roughness might impede precise spot-wise orientation mapping.

## Discussion

Ma et al.^10^ discussed the dependency of ablation rate regarding the fluence thresholds, in terms of the Gaussian intensity distribution along the laser spot. At sufficiently high overall laser power, considerable ablation takes place near the center of the laser spot, as the fluence is above the high fluence ablation threshold, surrounded by a less ablated zone, where the fluence is still above the low fluence threshold. When the overall power and thus intensity is reduced, prior high fluence areas might still show low fluence ablation and low fluence areas might show no significant ablation anymore. Therefore, the USP laser ablation process is generally described by multiple ablation thresholds, above which the ablation rate logarithmically increases with fluence, and which are generally material- and pulse-duration dependent. For copper and pulses with about 300 fs duration, the low fluence threshold was determined to be around 0.2 J/cm^2^ and the high fluence threshold around 1 J/cm^2^.^8,9^ If a certain number of pulses are applied above the low fluence ablation threshold, LIPSS develop uniformly along the exposed zone. This behavior is documented regarding different fluence values and PPS for perpendicular incident laser pulses, where generally few pulses are sufficient to create traceable LIPSS.^26,27,30,38^

For the interpretation of the acquired results and comparison with literature values, the special case of glancing-incident processing seems crucial. Since the trench inclination increases with respect to subsequently applied pulses, the fluence follows a cosine-related contrary trend for the projected laser spot on the processed surface (see Fig. 1b). Here, a trench taper of about 10° corresponds to a six-fold decrease in fluence.^42^ Furthermore, reflection tends to increase on an increasingly inclined surface and therefore additionally decreases the fluence at the surface.

Besides a considerably smaller ablation depth at the lowest fluence value of 1.30 J/cm^2^, as evaluates in Fig. S1, no explicit change in visual trench appearance was evident. The characterization concerning LIPSS formation yields a PPS threshold of about 18 k, respectively 25 k for the highest fluence level, above which LIPSS-covered areas tend to nucleate and grow with additional PPS applied. Notably, this threshold may also be related to the individual saturation taper values. As also discussed in Ref. 16, this is presumably related to the strong limitation of fluence for glancing-incident processing. In this case, the retarded LIPSS formation and outstanding roughness trend at the highest fluence level might be originating from a high fluence ablation threshold effect, which might still be present on a mildly inclined surface, where the fluence has already dropped below for lower levels. Also, the evolution of the taper (see Fig. S6) reflects a considerably increased ablation rate for the highest fluence value compared to those near 2 J/cm^2^.

The comparison of high and low fluence ablation might be of importance for the explanation of this circumstance since the ablation mechanism changes considerably. Consequently, it can be argued that, with increasing taper on the processed surface, the fluence declines to a specific point, where it drops below the high fluence ablation threshold, but the interaction in the low fluence regime still precedes. With that in mind, the whole process can be divided into two phases:

The first phase shows ongoing but declining material removal in the high fluence regime, acting up to about 18 k, respectively 25 k, PPS and leaving freshly ablated LIPSS free surface. Although a higher fluence enables a higher material removal at the onset of processing, the fluence in any case undergoes the cosine-related decline. Consequently, the saturation taper will be comparable, as depicted in Fig. S6, where even a factor of 2.7 in initial fluence is present between the trends. This is in accordance with the investigation of Sikora et al.,^7^ where a consistent similar taper evolution trend was present for all fluence levels, just in a different number of scan repetitions.

The second phase proceeds after the saturation taper is reached. When the processing continues, the interaction in the low fluence regime leads to the formation of LIPSS, nucleating from a confined area and spreading across the trench until the whole surface is covered. Local slightly less inclined surface parts might be the reason for the nucleation behavior, whereby the exact reason remains unclear. Furthermore, a considerably higher number of pulses is required for the formation in comparison to normal incident processing, presumably due to a strongly decreased local fluence. Nevertheless, this also holds true for the edge of the trenches, where LIPSS develop in any trench. In this region, the Gaussian tails of the pulse intensity lead to less ablation but to structuring in the lower fluence regime.

In principle, the suggested interdependency of the ablation process with the change in processed geometry should by itself be independent of the investigated material as long as the condition of massive bulk ablation is met at the initial phase of processing, corresponding to an initial processing fluence well above the high fluence ablation threshold. Further, the LIPSS formation would also have to follow the same fundamental principles. In our work so far, we can confirm this for metallic materials, and it would be interesting to expand this experimental confirmation to a wider range of material classes.

## Conclusion

The parameter investigation conducted within this work opens a perspective on the formation of unwanted LIPSS (laser-induced periodic surface structures), which might be avoided by an appropriate selection of parameters, in combination with a suitable processing pattern. The ablation of bulk material leads to strongly inclined surfaces, and with that to a drop of fluence below the high fluence ablation threshold at a certain point. Just before the saturation taper angle is reached, LIPSS are generated due to interaction in the low fluence regime upon subsequently repeated scanning of the obtained material surface. Consequently, if the pulse number is chosen accordingly (below a certain amount of cumulated laser pulses on a specific spot), no LIPSS are formed. To the best of our knowledge, the perspective of tuning process parameters to avoid surface patterning after the bulk ablation phase is presented in the current work for the first time. In the case of LIPSS-free surfaces, EBSD grain indexing can be applied directly for coarse- and fine-grained materials. However, even if LIPSS form, the material damage is very small, and the associated surface roughness can be easily removed via FIB if necessary.

## Supplementary Information

Below is the link to the electronic supplementary material.

Supplementary file 1 (PDF 2211 KB)
