# Supplementary material for: A Perspective to Control Laser-Induced Periodic Surface Structure Formation at Glancing-Incident Femtosecond Laser-Processed Surfaces
Source: JOM (1989). 2021 Nov 1;73(12):4248–57. doi: 10.1007/s11837-021-04963-w (PMC8666433; doi:10.1007/s11837-021-04963-w)
Supplement: Supplementary file 1 — (PDF 2211 KB) [file 11837_2021_4963_MOESM1_ESM.pdf]

Supplementary Material for:

A perspective to control laser-induced periodic surface structure formation at glancing incident femtosecond laser processed surfaces

Alexander Jelinek[1], Manuel J. Pfeifenberger[2], Reinhard Pippan[2], Daniel Kiener[1]

[1] Department of Materials Science, Chair of Materials Physics, Montanuniversität Leoben, Franz-Josef-Straße 18, 8700 Leoben, Austria

[2] Erich Schmid Institute for Materials Science, Austrian Academy of Sciences, Jahnstraße 12, 8700 Leoben, Austria

Corresponding Author: Alexander Jelinek, alexander.jelinek@unileoben.ac.at

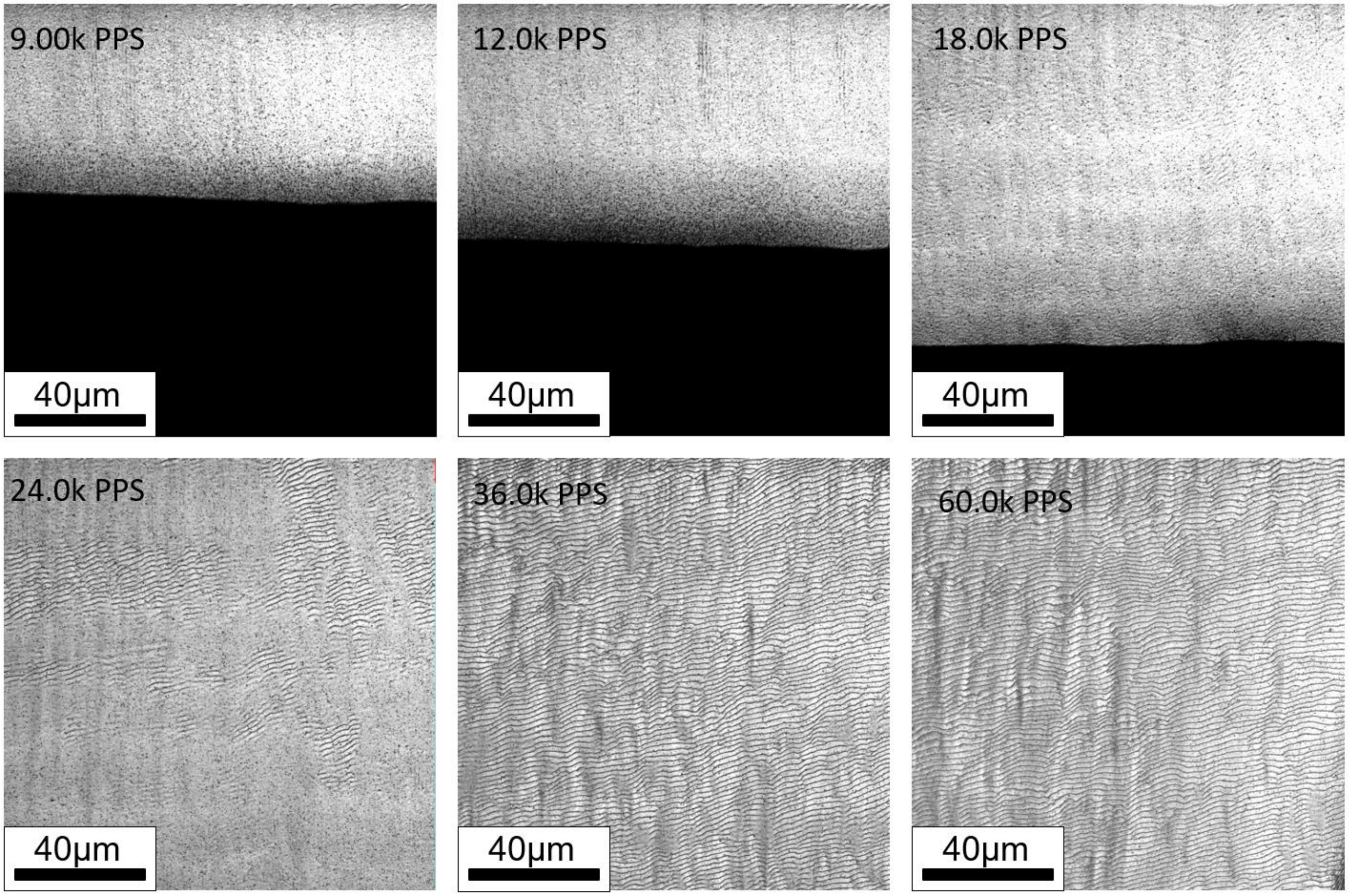

Fig. S1: LIPSS evolution of coarse-grained copper with different PPS settings, processed with a fluence of 1.30 J/cm². In the first three images the trenches are not deep enough to fill an area of 129 x 129 µm² scanned by the CLSM, therefore a reduced window of 65 x 65 µm² was applied for the surface roughness evaluation.

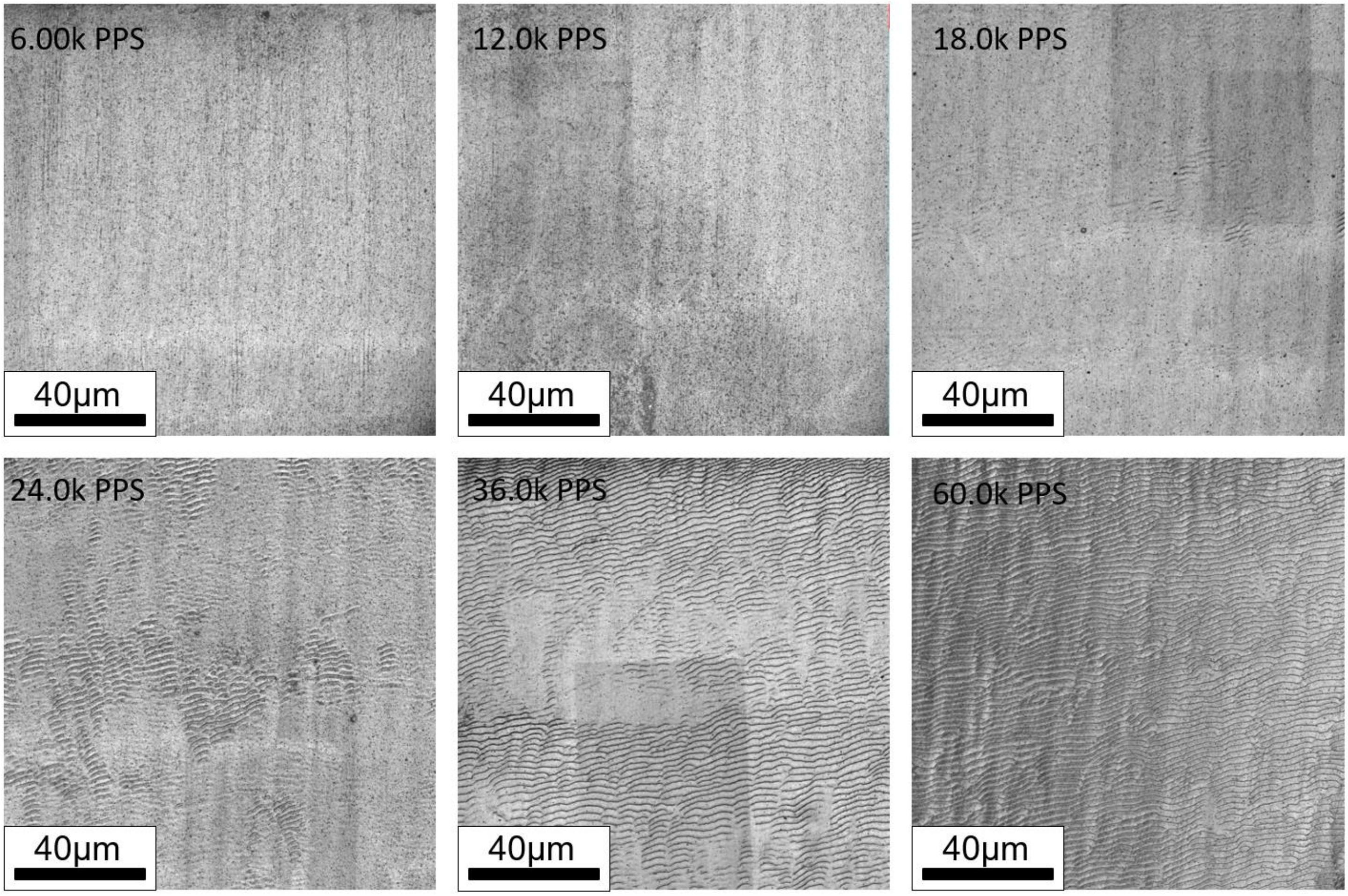

Fig. S2: CLSM images of coarse-grained copper with increasing PPS settings and processed with a fluence of 1.96 J/cm². Dark squares at individual images are remnants of SEM scans after processing. Within the 18k PPS trench, slight traces of LIPSS formation are present, indicating the onset of increasing roughness.

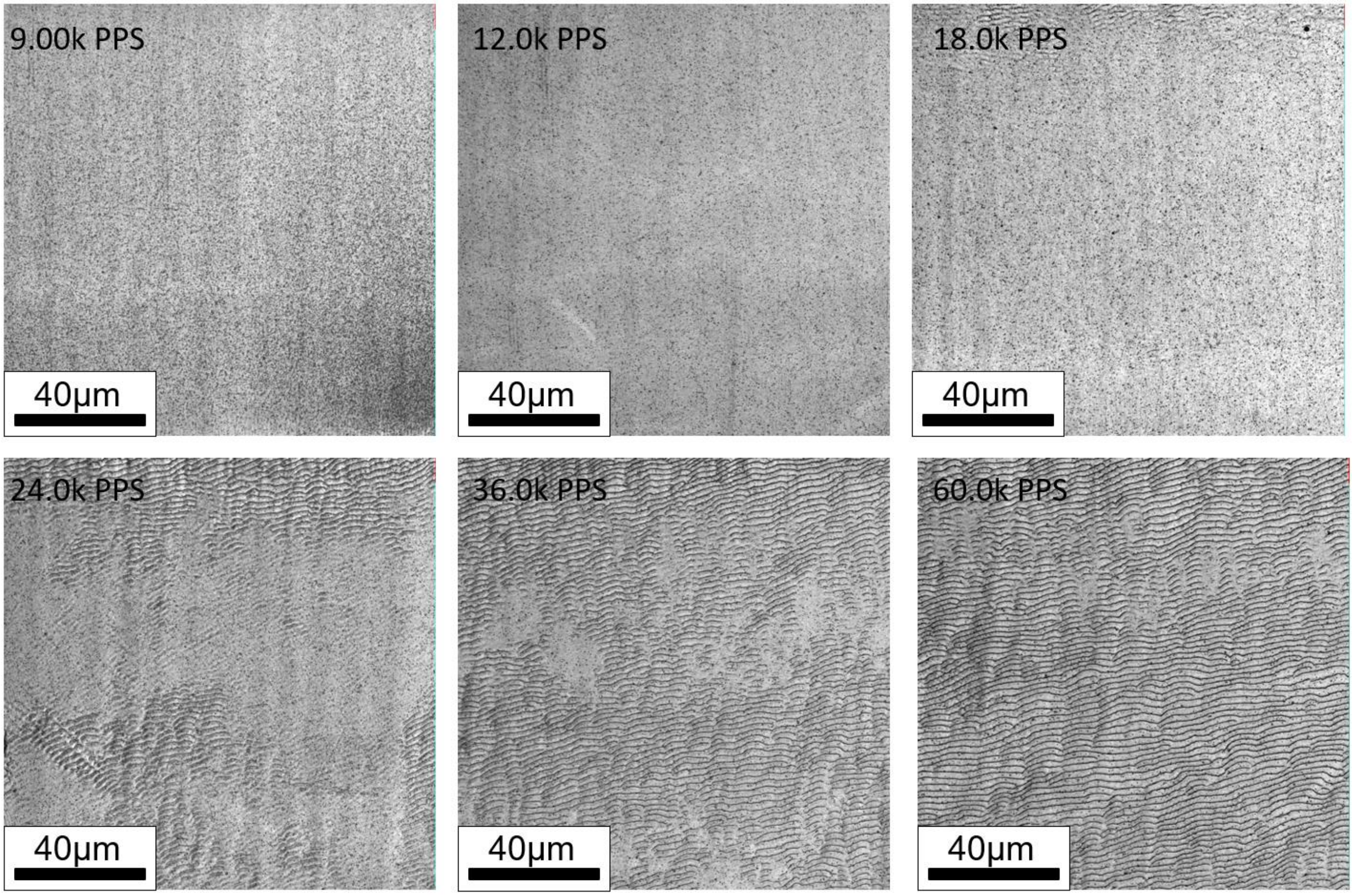

Fig. S3: Imaged trend of LIPSS evolution acquired by CLSM. For the processing of this trenches a fluence of 2.62 J/cm^2 was utilized. LIPPS in the third image of 18.0 kPPS are located near the upper edge of the trench. Outgoing from this area LIPSS fields develop, which is evident if compared with the fourth image, where the field has increased as well as new ones developed.

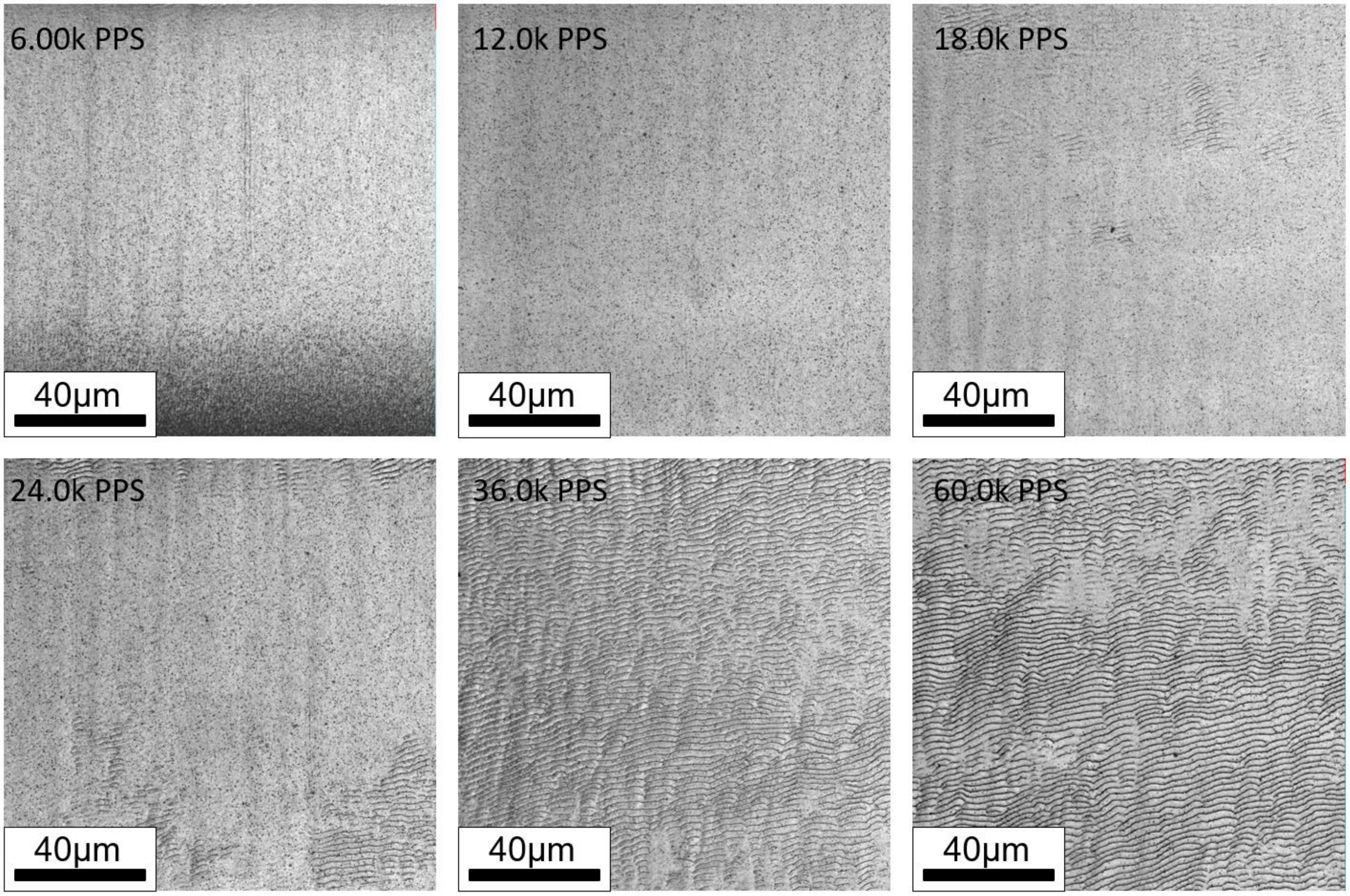

Fig. S4: The evolution of LIPSS is shown for increasing PPS, processed at a fluence level of 3.92 J/cm². Here LIPSS start to develop evidently at just below 18.0 kPPS in a spot wise manner at the upper regions of the trench.

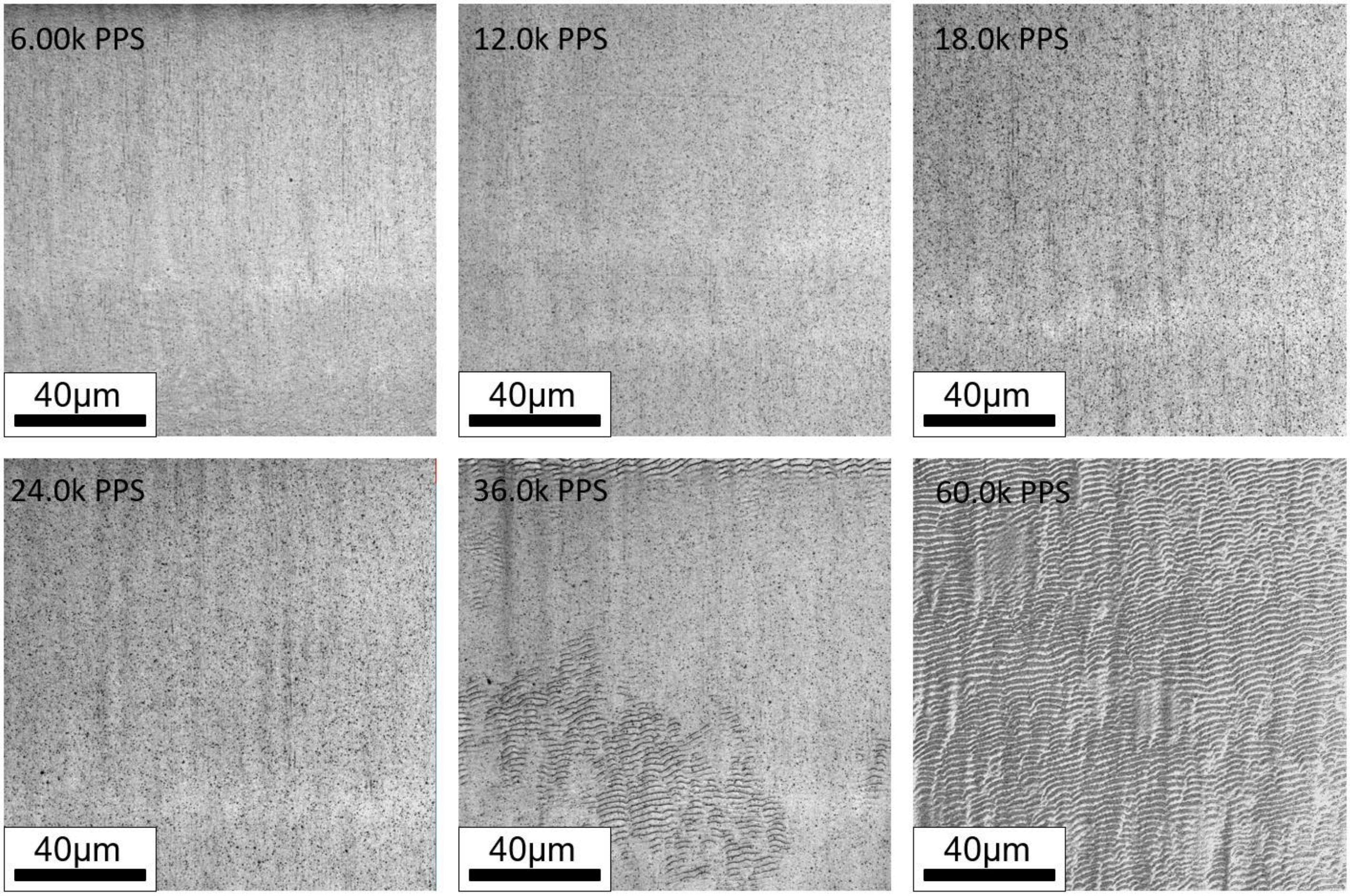

Fig. S5: At the highest fluence level of 5.24 J/cm² used for this study a different trend within the evolution of LIPSS on trenches occurred. Here no LIPSS are present at 18.0k PPS, as for lower fluence levels (see Fig. 7, 8, 9 and 10) and even not at 24.0 PPS. Although the evolution is retarded in terms of applied PPS, the field wise evolution (see image with 36.0 kPPS) trend appears to be comparable and the final surface (see last image of 60.0 kPPS) look similar to them of lower energy levels.

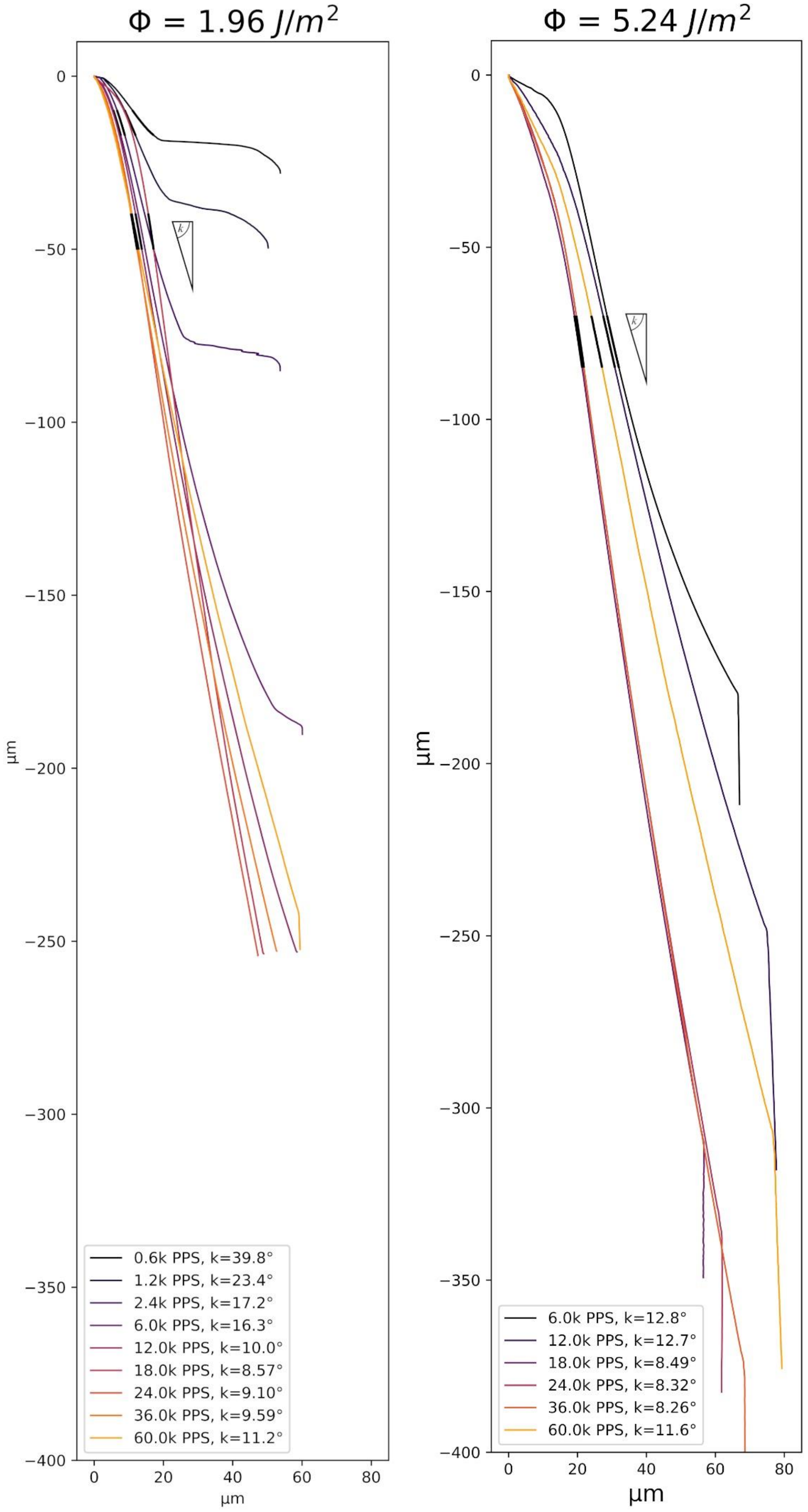

Fig. S6: CLSM height profiles, acquired at the center of the trenches for two fluence levels and with increasing PPS count. The evolution of the trench cross section is shown, obtained by gracing incident laser processing (from the top). The zero-point indicates the original surface, while the strong decline at the end of individual graphs corresponds to the perpendicular face. The evolution of the trench taper *k* is linearly fitted in the flat region (black marking) and evaluated with respect to the vertical direction.

Tab. S1: All parameter combinations applied along all processed trenches, datapoints with equal PPS and fluence number are marked as duplicates. The appearance of LIPSS was evaluated visually via SEM images.

**Nr**	**frequency**	**divisor**	**spot diameter**	**scanning speed**	**lines**	**scans**	**line dist.**	**PPS**	**power**	**Fluence**	**LIPSS**	**Duplicate**
-	*f*	*div*	*D*	*s*	*l*	*scans*	a_l	-	*P*	-	-	0 = no
-	Hz	-	m	m/s	-	-	m	-	mW	J/cm²	-	1 = yes
**1**	50000	1	0.0000245	0.001	10	10	0.00001	300125	1.54	0.65	yes	0
**2**	50000	1	0.0000245	0.001	10	10	0.00001	300125	2.16	0.98	yes	0
**3**	50000	1	0.0000245	0.001	10	10	0.00001	300125	3.09	1.31	yes	0
**4**	50000	1	0.0000245	0.001	10	10	0.00001	300125	4.63	1.96	yes	0
**5**	50000	10	0.0000245	0.001	10	10	0.00001	30012.5	1.54	1.05	yes	0
**6**	50000	10	0.0000245	0.001	10	10	0.00001	30012.5	2.16	0.98	yes	0
**7**	50000	10	0.0000245	0.001	10	10	0.00001	30012.5	3.09	1.31	yes	0
**8**	50000	10	0.0000245	0.001	10	10	0.00001	30012.5	4.63	1.96	yes	0
**9**	50000	50	0.0000245	0.001	10	10	0.00001	6002.5	1.54	1.57	no	0
**10**	50000	50	0.0000245	0.001	10	10	0.00001	6002.5	2.16	0.98	no	1
**11**	50000	50	0.0000245	0.001	10	10	0.00001	6002.5	3.09	1.31	no	1
**12**	50000	50	0.0000245	0.001	10	10	0.00001	6002.5	4.63	1.96	no	1
**13**	50000	1	0.0000245	0.001	10	1	0.000005	60025	1.54	2.10	yes	1
**14**	50000	1	0.0000245	0.001	10	1	0.000005	60025	2.16	0.98	yes	1
**15**	50000	1	0.0000245	0.001	10	1	0.000005	60025	3.09	1.31	yes	1
**16**	50000	1	0.0000245	0.001	10	1	0.000005	60025	4.63	1.96	yes	1
**17**	50000	10	0.0000245	0.001	10	10	0.000005	60025	1.54	2.62	yes	0
**18**	50000	10	0.0000245	0.001	10	10	0.000005	60025	2.16	0.98	yes	0
**19**	50000	10	0.0000245	0.001	10	10	0.000005	60025	3.09	1.31	yes	0
**20**	50000	10	0.0000245	0.001	10	10	0.000005	60025	4.63	1.96	yes	0
**21**	50000	50	0.0000245	0.001	10	10	0.000005	12005	3.09	1.31	no	1
**22**	50000	50	0.0000245	0.001	10	10	0.000005	12005	4.63	1.96	no	1
**23**	50000	50	0.0000245	0.001	10	10	0.000005	12005	6.18	2.62	no	1
**24**	50000	10	0.0000245	0.005	10	10	0.000005	12005	2.16	0.98	no	0
**25**	50000	10	0.0000245	0.005	10	10	0.000005	12005	3.09	1.31	no	1
**26**	50000	10	0.0000245	0.005	10	10	0.000005	12005	4.63	1.96	no	1
**27**	50000	10	0.0000245	0.005	10	10	0.000005	12005	6.18	2.62	no	1
**28**	50000	100	0.0000245	0.001	10	10	0.000005	6002.5	2.16	0.98	no	0
**29**	50000	100	0.0000245	0.001	10	10	0.000005	6002.5	3.09	1.31	no	1
**30**	50000	100	0.0000245	0.001	10	10	0.000005	6002.5	4.63	1.96	no	1
**31**	50000	100	0.0000245	0.001	10	10	0.000005	6002.5	6.18	2.62	no	1
**32**	50000	20	0.0000245	0.005	10	10	0.000005	6002.5	3.09	1.31	no	0
**33**	50000	20	0.0000245	0.005	10	10	0.000005	6002.5	4.63	1.96	no	1
**34**	50000	20	0.0000245	0.005	10	10	0.000005	6002.5	4.63	1.96	no	1
**35**	50000	20	0.0000245	0.005	10	10	0.000005	6002.5	6.18	2.62	no	1
**36**	50000	50	0.0000245	0.001	1	100	0.000005	12005	4.63	1.96	no	1
**37**	50000	50	0.0000245	0.001	100	1	0.000005	12005	4.63	1.96	no	1
**38**	50000	50	0.0000245	0.001	10	10	0.000005	12005	12.35	5.24	no	1
**39**	50000	50	0.0000245	0.007	100	1	0.000001	8575	12.35	5.24	no	0
**40**	50000	20	0.0000245	0.005	10	10	0.000005	6002.5	6.18	2.62	no	0
**41**	50000	50	0.0000245	0.001	10	10	0.000008	7503.13	4.63	1.96	no	0
**42**	50000	50	0.0000245	0.001	1	1	0.000005	120.05	4.63	1.96	no	0
**43**	50000	50	0.0000245	0.001	1	2	0.000005	240.1	4.63	1.96	no	0

**44**	50000	50	0.0000245	0.001	1	5	0.000005	600.25	4.63	1.96	no	0
**45**	50000	50	0.0000245	0.001	1	10	0.000005	1200.5	4.63	1.96	no	0
**46**	50000	50	0.0000245	0.001	1	20	0.000005	2401	4.63	1.96	no	0
**47**	50000	1	0.0000245	0.001	3	2	0.000005	36015	4.63	1.96	yes	1
**48**	50000	5	0.0000245	0.001	3	10	0.000005	36015	4.63	1.96	yes	1
**49**	50000	10	0.0000245	0.001	3	20	0.000005	36015	4.63	1.96	yes	1
**50**	50000	15	0.0000245	0.001	3	30	0.000005	36015	4.63	1.96	yes	1
**51**	50000	20	0.0000245	0.001	3	40	0.000005	36015	4.63	1.96	yes	1
**52**	50000	25	0.0000245	0.001	3	50	0.000005	36015	4.63	1.96	yes	1
**53**	50000	30	0.0000245	0.001	3	60	0.000005	36015	4.63	1.96	yes	1
**54**	50000	40	0.0000245	0.001	3	80	0.000005	36015	4.63	1.96	yes	1
**55**	50000	35	0.0000245	0.001	3	70	0.000005	36015	4.63	1.96	yes	1
**56**	50000	45	0.0000245	0.001	3	90	0.000005	36015	4.63	1.96	yes	0
**57**	50000	50	0.0000245	0.001	3	100	0.000005	36015	4.63	1.96	yes	1
**58**	50000	55	0.0000245	0.001	3	110	0.000005	36015	4.63	1.96	yes	1
**59**	50000	100	0.0000245	0.001	3	200	0.000005	36015	4.63	1.96	yes	1
**60**	50000	50	0.0000245	0.001	1	50	0.000005	6002.5	4.63	1.96	no	0
**61**	50000	50	0.0000245	0.001	1	100	0.000005	12005	4.63	1.96	no	0
**62**	50000	50	0.0000245	0.001	1	200	0.000005	24010	4.63	1.96	yes	0
**63**	50000	50	0.0000245	0.001	10	10	0.000005	12005	6.18	2.62	no	0
**64**	50000	50	0.0000245	0.001	10	10	0.000005	12005	9.26	3.93	no	1
**65**	50000	50	0.0000245	0.001	1	125	0.000005	15006.25	4.63	1.96	no	0
**66**	50000	50	0.0000245	0.001	1	150	0.000005	18007.5	4.63	1.96	yes	0
**67**	50000	50	0.0000245	0.001	1	300	0.000005	36015	4.63	1.96	yes	0
**68**	50000	10	0.0000245	0.001	1	20	0.000005	12005	4.63	1.96	no	1
**69**	50000	2	0.0000245	0.005	1	20	0.000005	12005	4.63	1.96	no	0
**70**	50000	50	0.0000245	0.001	1	100	0.000005	12005	3.09	1.31	no	1
**71**	50000	50	0.0000245	0.001	1	100	0.000005	12005	9.26	3.93	no	0
**72**	50000	50	0.0000245	0.001	1	100	0.000005	12005	12.35	5.24	no	0
**89**	50000	50	0.0000245	0.001	1	150	0.000005	18007.5	6.18	2.62	no	0
**90**	50000	50	0.0000245	0.001	1	150	0.000005	18007.5	12.35	5.24	no	0
**91**	50000	50	0.0000245	0.001	1	200	0.000005	24010	6.18	2.62	yes	0
**92**	50000	50	0.0000245	0.001	1	200	0.000005	24010	9.26	3.93	yes	0
**93**	50000	50	0.0000245	0.001	1	200	0.000005	24010	12.35	5.24	yes	0
**94**	50000	50	0.0000245	0.001	1	200	0.000005	24010	3.09	1.31	yes	1
**95**	50000	50	0.0000245	0.001	1	300	0.000005	36015	6.18	2.62	yes	0
**96**	50000	50	0.0000245	0.001	1	300	0.000005	36015	12.35	5.24	yes	0
**97**	50000	50	0.0000245	0.001	1	500	0.000005	60025	6.18	2.62	yes	0
**98**	50000	50	0.0000245	0.001	1	500	0.000005	60025	9.26	3.93	yes	0
**99**	50000	50	0.0000245	0.001	1	75	0.000005	9003.75	3.09	1.31	no	0
**100**	50000	50	0.0000245	0.001	1	100	0.000005	12005	3.09	1.31	no	0
**101**	50000	50	0.0000245	0.001	1	150	0.000005	18007.5	3.09	1.31	no	0
**102**	50000	50	0.0000245	0.001	1	200	0.000005	24010	3.09	1.31	yes	0
**103**	50000	50	0.0000245	0.001	1	300	0.000005	36015	3.09	1.31	yes	0
**104**	50000	50	0.0000245	0.001	1	75	0.000005	9003.75	6.18	2.62	no	0
**105**	50000	50	0.0000245	0.001	1	50	0.000005	6002.5	9.26	3.93	no	0
**106**	50000	50	0.0000245	0.001	1	50	0.000005	6002.5	12.35	5.24	no	0
